# Biphasic Polyurethane/Polylactide Sponges Doped with Nano-Hydroxyapatite (nHAp) Combined with Human Adipose-Derived Mesenchymal Stromal Stem Cells for Regenerative Medicine Applications

**DOI:** 10.3390/polym8100339

**Published:** 2016-10-11

**Authors:** Krzysztof Marycz, Monika Marędziak, Jakub Grzesiak, Anna Lis, Agnieszka Śmieszek

**Affiliations:** 1 Electron Microscopy Laboratory, Wroclaw University of Environmental and Life Sciences, Wroclaw 51-631, Poland; smieszek.agnieszka@gmail.com; 2 Department of Animal Physiology and Biostructure, Wroclaw University of Environmental and Life Sciences, Wroclaw 50-375, Poland; monika.maredziak@gmail.com; 3 Electron Microscopy Laboratory, Wroclaw Research Centre EIT+, Wroclaw 54-066, Poland; grzesiak.kuba@gmail.com; 4 Faculty of Materials Science and Ceramics, AGH University of Science and Technology, Krakow 30-059, Poland; annal@agh.edu.pl

**Keywords:** thermoplastic polyurethane, polylactide, nano-hydroxyapatite, adipose stem cells, biomaterials

## Abstract

Cartilage and bone tissue injuries are common targets in regenerative medicine. The degeneration of cartilage tissue results in tissue loss with a limited ability to regenerate. However, the application of mesenchymal stem cells in the course of such condition makes it possible to manage this disorder by improving the structure of the remaining tissue and even stimulating its regeneration. Nevertheless, in the case of significant tissue loss, standard local injection of cell suspensions is insufficient, due to the low engraftment of transplanted cells. Introduction of mesenchymal stem cells on the surface of a compatible biomaterial can be a promising tool for inducing the regeneration by both retaining the cells at the desired site and filling the tissue gap. In order to obtain such a cell-biomaterial hybrid, we developed complex, biphasic polymer blend biomaterials composed of various polyurethane (PU)-to-polylactide (PLA) ratios, and doped with different concentrations of nano-hydroxyapatite (nHAp). We have determined the optimal blend composition and nano-hydroxyapatite concentration for adipose mesenchymal stem cells cultured on the biomaterial. We applied biological in vitro techniques, including cell viability assay, determination of oxidative stress factors level, osteogenic and chondrogenic differentiation potentials as well as cell proteomic analysis. We have shown that the optimal composition of biphasic scaffold was 20:80 of PU:PLA with 20% of nHAp for osteogenic differentiation, and 80:20 of PU:PLA with 10% of nHAp for chondrogenic differentiation, which suggest the optimal composition of final biphasic implant for regenerative medicine applications.

## 1. Introduction

Cartilage and bone tissue injuries caused by mechanical factors require surgical intervention due to their sudden and unexpected nature. These disorders are caused by a single or multiple traumatic events that usually lead to serious motion limitation, pain, limb stiffness and/or swelling, which seriously reduce the life quality, particularly in elderly patients. Lesions of both articular cartilage and subchondral part of the bone requires the use of regenerative medicine instruments as well as tissue engineering solutions to fit these different in structure and function tissues as one multicompatible bioimplant [1]. Articular cartilage has a low capacity for self-renewal and limited regeneration potential when injured. Cartilage is largely avascular tissue, which generally significantly impairs the regeneration process [2]. Various methods have been proposed for cartilage and bone regeneration, including transplantation of osteochondral autologous graft, cultured chondrocytes suspended in a biodegradable scaffold and/or 3D biphasic scaffolds [3,4,5]. Currently, a promising therapeutic strategy for the treatment of these two tissues is the application of biodegradable and biocompatible 3D scaffold that will enhance the regeneration of both injured tissues, i.e., articular cartilage and bone. Synthetic biomaterials composed of polyurethane (PU) and a polymer of lactic acid (PLA) have been recently proposed as an engineered scaffold dedicated for regenerative medicine applications due to their biocompatibility, relative ease of modulation of their microstructure and mechanical properties [6]. Additionally, it was recently demonstrated that PU/PLA-based biomaterials released nontoxic degradation products, which did not reduce cellular viability. Moreover, our recent findings have indicated that the PU/PLA blends supports adhesion ofboth olfactory ensheathing glial cells (OECs) as well as adipose-derived mesenchymal stromal stem cells (ASC) in vitro, maintaining their proper morphology and behavior [7]. In turn, hydroxyapatite (HAp) is a highly desirable material in bone regeneration field due to its high biocompatibility. HAp can be obtained from different types of sources, e.g. directly from the patient (autologous), another human donor (allogenic), animals (xenogenic) as well as by synthetic production [8]. It is well known, however, that synthetic hydroxyapatites exhibit very low osteoconductive and osteoinductive potentials [9]. Natural HAps in the bones or/and teeth occur in the form of nanocrystals, which is a prerequisite for their internalization by tissues, and this feature makes natural HAp more effective during the replacement therapy [10]. Therefore the synthetic HAp designed for bone treatment should be prepared in a form of nanocrystals. Hence, preparation of such small objects using special synthetic methods provide appropriate equilibrium in the resorption-remineralization cycle and a high affinity for proteins that play an important role in the active forming of osseous and fibrous tissues [11].

In recent years, regenerative medicine involving stem cell therapies became a promising therapeutic tool, especially in combination with resorbable biocompatible scaffolds. Many research and clinical groups showed the beneficial effect of mesenchymal stromal stem cells (MSCs) as therapeutic agents both in vitro and in vivo [12,13]. MSCs are a widespread group of cells in the body present in most of stromal tissues, which can be easily isolated and propagated in vitro. They are characterized bythe ability to self-renew and differentiate in vitro into multiple lineages, including osteogenic, chondrogenic and adipog enic. Regardless their tissue of origin, they share similar molecular phenotype CD44^+^/CD73^+^/CD90^+^/CD105^+^/CD34^−^/CD45^−^ [14]. In addition to proliferative and differentiation potentials, MSCs are well known for their immunomodulatory and immunosuppressive properties, which make them even more promising tool in regenerative medicine [15]. The pro-regenerative potential of MSCs is explained by their paracrine activity and the ability to deliver locally membrane derived vesicles (MVs) and exosomes, rich in a broad range of growth factors and regulatory RNAs. Although the role of membrane vesicles and exosomes in the course of regenerative process is still poorly described, it seems that they transfer to target cells molecules, such as proteins, lipids, RNAs and cytokines, including transforming growth factors (TGFs), bone morphogenic proteins (BMPs), or vascular endothelial growth factors (ExoCarta database contains a comprehensive list of proteins, lipids, and RNAs associated with microvesicles) [16,17,18].

In order to acquire the most optimal composition of the polymer scaffold, designed as a mesenchymal stem cell carrier for future clinical applications, an in vitro evaluation was performed that focused on MSC osteogenic and chondrogenic differentiation potentials on different compositions of materials, as reliable markers of biocompatibility with bone and cartilage tissues. In the present study, we have found that the PU/PLA ratio and incorporation of nHAp affected ASC behavior, viability and differentiation potential. We have determined an optimal structure of 3D biphasic scaffold, which is composed of the PU/PLA (20:80) sponge enriched with 20% of nHAp on the bone-site side, and the PU/PLA (80:20) sponge enriched with 10% of nHAp on the cartilage-site side.

## 2. Materials and Methods

### 2.1. Materials

The following reactants were used: synthetic hydroxyapatite nanoparticles (nHAp) (Ca_5_(OH)(PO_4_)_3_, <100 nm. NanoSynHAp, Poznan, Poland), sodium chloride as porogen (NaCl particle size: 300 ÷ 600 µm, POCH S.A., Gliwice, Poland), thermoplastic polyurethane (TPU) (Elastollan^®^ A12P000-INTiBS PAN, Wroclaw, Poland), inelastic polylactide PURASORB^®^ (PLA) (PURACbiochembv, Gorinchem, The Netherlands), dimethyloformamide (DMF) as a solvent in molecular biology (≥99.0% purity, Sigma Aldrich, Poznan, Poland), and sterile filtered water for molecular biology methods and cell culture (Sigma Aldrich).

### 2.2. Preparation of Biomaterials

#### 2.2.1. Preparation of Polymer Nanocomposite Thin Films

**Scheme 1:**
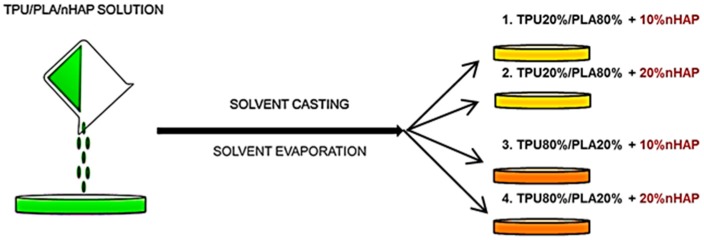
Schematic illustration of the solvent casting method used for TPU/PLA/HAP nanocomposite fabrication destined for biological evaluation.

In the present study, TPU/PLA/nHAp thin films were prepared on glass Petri dishes by solvent casting (Scheme 1) for in vitro experiments.

Polymer solutions were prepared at 5% concentration (*w*/*v*). Polymers were dissolved in DMF by magnetic stirring for 72 h at 45 °C. For the bioceramic HAp nanoparticles doping, the TPU/PLA solution was mixed with the nHAp powder at various concentrations (Table 1). The amounts of hydroxyapatite added to the TPU/PLA solution were 10% and 20% *w*/*v*. The resulting solutions were stirred with a magnetic stirrer at 45 °C for 24 h and an ultrasonic stirrer for 3 h at room temperature to obtain homogenous solutions. The prepared polymer/nHAp solutions (20 mL) were casted into glass Petri dishes (ø 100 mm). After DMF evaporation in air atmosphere at room temperature (12 h) and under vacuum at 30 °C (12 h), the casted nanocomposite films were extracted from Petri dishes. The remaining DMF on the composite films was removed using filtered distilled water. The TPU/PLA/nHAp nanocomposite thin films were rinsed in filtered distilled water and dried in air and under vacuum at room temperature before the plasma sterilization procedure. All films were then put into the TYVEK sterile packages (EN 868/ISO 11607, H_2_O_2_ plasma, Sigma Aldrich, Poznan, Poland). The prepared nanocomposite films were sterilized by the low temperature plasma sterilization method with a vaporized hydrogen peroxide prior to their in vitro biological evaluation. Four different types of TPU/PLA/nHAp nanocomposites were prepared from polyurethane, polylactide and hydroxyapatite nanoparticles for this analysis.

#### 2.2.2. Preparation of Porous Polymer Nanocomposite Scaffold and Bi-Phasic Implant

**Scheme 2:**
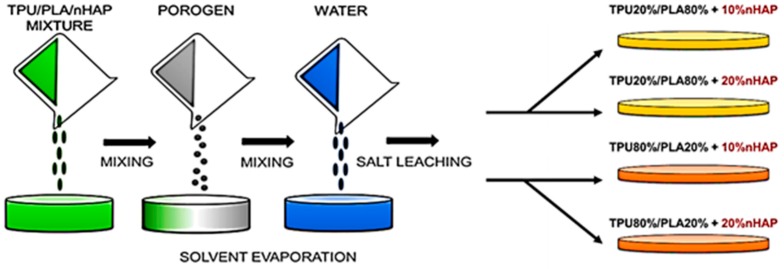
Schematic illustration of the solvent casting/salt leaching method used for porous TPU/PLA/HAP nanocomposite fabrication destined for physical evaluation.

**Scheme 3:**
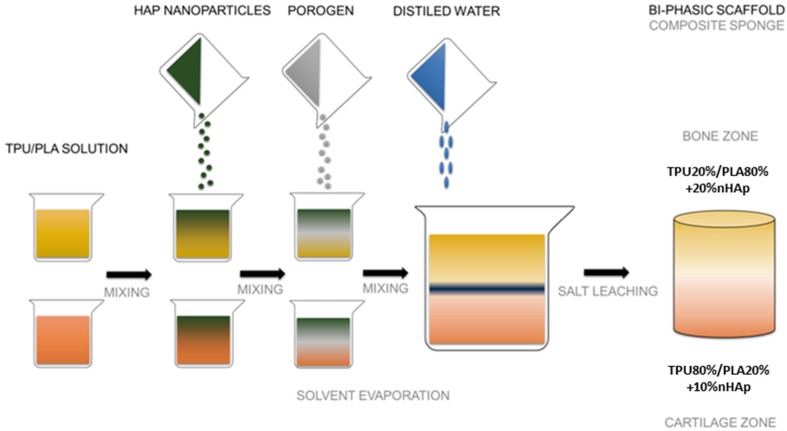
Schematic illustration of the solvent casting/salt leaching method used for TPU/PLA/nHAp biphasic scaffold fabrication.

Porous TPU/PLA/nHAp nanocomposite sponges and bi-phasic implants made from them were prepared by the conventional solvent casting/salt leaching (SCSL) method (Scheme 2 and Scheme 3) for physical evaluation. The polymer granules were dissolved in DMF and stirred for 72 h at 45 °C to prepare a concentration of 10% *w*/*v* solution. The resulting homogenous solution was used to prepare the pre-form. The TPU/PLA solution was mixed with nHAp particles of different concentrations (Table 2). The proportion of nHAp to TPU/PLA solutions was 10% and 20% *w*/*v*. The resulting polymer/nHAP solution was stirred with magnetic stirrer at 45 °C for one day and ultrasonic stirrer for 3 h at room temperature to obtain a homogenous solution. The polymer/nHAP solution was mixed with sieved sodium chloride grains (300–600 µm). The mixture was stirred at room temperature for 30 min. Then the TPU/PLA/nHAP/salt mixture in DMF was poured into 50 mL glass beakers and kept for 1 h under a fume hood. Excess polymer solution on the top surface was removed using micropipettes. The formed polymer/nHAp/porogen mixture in the glassware was air-dried for 120 h, and then vacuum-dried for 48 h, followed by salt leaching in distilled water until the pH was nearly 7. As a result, three-dimensional porous nanocomposites sponges were produced. The final form of the nanocomposite sponges were air-dried and vacuum-dried for 24 h.

### 2.3. Characterization of Biomaterials

#### 2.3.1. SEM/EDX Analysis

Characterization of the morphology and chemical elements of the TPU/PLA/HAp composite films and sponges was examined on a scanning electron microscope (SEM, Zeiss EVO LS15, Oberkochen, Germany). For SEM investigation, nanocomposite samples were coated with gold (ScanCoat6, Edwards, Warszawa, Poland). Cross-section and external top and bottom surfaces of the nanocomposite samples were fixed on SEM sample holders using conductive carbon tape.

#### 2.3.2. Profilometry

The roughness of the obtained TPU/PLA/nHAp composite films was analysed by a profilometer (Hommelwerke, Schwenningen, Germany). The roughness of 10 different spots on both sides of each sample was determined and the average surface roughness (*R*_a_) was evaluated. All values are presented as means of ten measurements (±standard deviation (SD)).

#### 2.3.3. 3D Analysis, BET Analysis—Porosity

Total porosity was determined using direct methods (determining the bulk volume of the porous sample, and then determining the volume of skeletal material with no pores (pore volume = total volume − material volume).

#### 2.3.4. Water Adsorption Capacity and Wettability

Contact angles of the films to water were measured on the air-surface of the samples using a DSA10 contact angle meter (Krüss, Hamburg, Germany). Water absorption of the polymeric composite sponge were measured by immersing the sponges in distilled water for a predetermined time period, then the films were taken out with filter paper vacuum-drier and finally weighed. To evaluate the hydrophilicity of the TPU/PLA blend doped with nHAp, their surface contact angle to water and water absorption were measured and compared to PLA and TPU. Drops of filtered distilled water of 2 µL volume were added using a syringe. The values of the water contact angle were obtained by averaging the values of at least ten measurements for each nanocomposite film. The wetting behaviour of the TPU/PLA/nHAp sponge was analysed by measuring the water adsorption capacity of the composite sponge in the air. The polymer sponges were immersed in distilled water for seven days. Wettability measurements were conducted six times (after 2, 24, 48, 72, 96 and 168 h). The water volume was 20 mL.

#### 2.3.5. Mechanical Properties—Tensile and Compressive Strength

Mechanical properties of the composite films (rectangular samples: 60 mm × 5 mm × 1 mm) were determined by measuring their tensile strength (σ) on a ZWICK Universal Test Machine with a tensile speed of 10 mm/min at room temperature.

#### 2.3.6. Mechanical Compressive (Sponge) and Tensile Test (Film)

A universal machine (1435, Zwick & Roell, Wroclaw, Poland) was used for the measurement of the compression and tensile strength of the nanocomposite materials. Cylindrically (*d* and *h* = 10 mm) shaped samples were prepared for mechanical testing. Cylindrical samples were compressed between parallel plates using fully dried samples. Compressive strain-stress curves of all sponge series were determined with a 10 N load cell and a cross-head speed of 1 mm/min. The compressive Young’s modulus (MPa) was calculated between 0.5–1 N based on the stress-strain curve. All values are means of ten measurements (±standard deviation).

#### 2.3.7. Thermal Analysis of TPU/PLA/nHAp Composites

The thermal degradation behaviour of TPU, PLA, TPU/PLA blends and their hydroxyapatite nanocomposites was investigated by termogravimetry (TGA) and differential scanning calorimetry (DSC). Thermal analyses of filler-free TPU/PLA blends and TPU/PLA/nHAP composites were carried out using a NETZSCH STA (449 F3 Jupiter, Selb, Germany) instrument under nitrogen. Initial sample weight was set as 5 mg for each operation. The specimen was heated from 20 to 600 °C at a heating rate of 10 °C/min.

#### 2.3.8. Focused Ion Beam—Scanning Electron Microscopy

In order to examine the presence of nHAp in the prepared polymer blends, focused ion beam (FIB) milling and low voltage scanning electron microscopy with energy-selective backscattered detector (EsB, Auriga 60, Zeiss, Oberkochen, Germany) were used. The samples were embedded in a low viscosity resin (Agar, Cambridge, UK). Next, the sample blocks were trimmed using a glass knife to recover the samples on the block surfaces and initially polish the examined surface. The prepared blocks were mounted on microscope stubs using carbon tape, sputtered with a 30-nm-thick layer of gold and placed in a microscope. The region of interest was localized, and a groove was milled with FIB in the selected sites using a 30 kV/4 nA aperture. The recovered surface was polished using a 30 kV/600 pA aperture. Polished surfaces were imaged at 2 kV using an EsB detector, with detector’s grid set to 0 V. Nano-hydroxyapatite particles were imaged and additionally examined using EDX mapping at 20 kV (Oxford, UK) to confirm the elemental composition of the nanoparticles.

#### 2.3.9. Isolation of Cells

All experiments were approved by the Second Local Ethics Committee at the Wroclaw University of Environmental and Life Sciences (registry number KB-177/2014, March 2014).

Adipose stem cells were isolated from subcutaneous fat tissue donated by patients aged 44–77 years during surgical procedures at the Jagiellonian University Medical College. Isolation procedures were performed according to the method described previously [19]. Removed tissue fragments were placed in sterile Hank’s balanced salt solution (HBSS, Sigma Aldrich) and transported to the laboratory. The material was minced with a surgical blade, washed extensively off of the blood traces in fresh HBSS, and placed in collagenase solution (5 mg/mL) in Dulbecco’s modified eagle medium/Nutrient mixture F12 Ham (DMEM F12, Sigma Aldrich) for 40 min at 37 °C/5% CO_2_, with extensive shaking every 10 min. The digested tissue was then centrifuged at 1200× *g* for 10 min, after which the supernatant with released oil and remaining tissue was discarded. Cell pellet was suspended in DMEM F12 containing 10% fetal bovine serum (FBS, Sigma Aldrich) and 1% antibiotics (penicillin/streptomycin/amphotericin b, PSA, Sigma Aldrich) and cultured in a humidified incubator at 37 °C/5% CO_2_ until cells reached about 80% confluence. Prior to the experiment, the cells from the primary cultures were further propagated in the secondary cultures up to three passages in Dulbecco’s modified eagle’s medium (DMEM, Sigma Aldrich) containing 4500 mg/L of glucose, 10% FBS and 1% PSA.

#### 2.3.10. Flow Cytometry

The immunophenotype analysis was performed by means of four-coloured cytofluorometry using a FACS Calibur flow cytometer (BD, Warszawa, Poland). For analysis, the following monoclonal, fluorophore-conjugated antibodies were used: CD34-PE, CD44-PE, CD45-PE, CD90-FITC, CD105-PerCP, CD73-APC (BD). The cells were detached from the culture vessels using accutase (BioWest, Nuaillé, France), followed by rinsing in PBS with 2% FBS at a concentration of 5 × 10^5^/mL. The cells were centrifuged at 300× *g* for 5 min, and the cell pellet was resuspended in a fresh culture medium containing proper antibodies (1:1000 dilution) and incubated at 4 °C for 20 min. The samples were washed three times in PBS and measured. At least 1 × 10^4^ of the cells were measured for each sample. The obtained results were analysed using the CellQuest Pro software (BD).

#### 2.3.11. Biomaterial in Vitro Evaluation

To evaluate the influence of biomaterials on adipose stem cells, the scaffolds were placed in a 24-well culture plate. Different types of biomaterials were used in this experiment, assigned according to Table 3 below.

Cells were seeded on biomaterials at a concentration of 3 × 10^4^ cells per well and cultured in DMEM F12 supplemented with 10% FBS and 1% PSA. After 48 h of culture, the media were replaced with differentiation-stimulating media specific for chondrogenic and osteogenic lineages (StemPro, Thermo Scientific, Waltham, MA, USA). The media were replaced every 72 h. Prior to the evaluation, the cells were maintained in culture conditions for 21 days. Control cells were seeded in a 24-well plate to empty wells. After the last day of the experiment, conditioned culture media were collected for further analysis. The cells for microscopic observations were fixed with biomaterials in 4% paraformaldehyde, while the cells for gene expression analysis were homogenized using TRIzol reagent (Thermo Scientific).

#### 2.3.12. Viability Assay

The bromodeoxyuridine assay (BrdU Cell Proliferation ELISA Kit, Abcam, Cambridge, UK) was performed to evaluate the viability and proliferative potential of cells cultured on different biomaterials and in control conditions. The test based on quantitative measurements of DNA synthesis was performed on the last day of the experiment, according to the standard protocol delivered with the kit. The intensity of immunoenzymatic reaction was measured with a spectrophotometric microplate reader (BMG Labtech, Ortenberg, Germany) at 450 and 550 nm wavelengths.

#### 2.3.13. Quantitative Analysis of Cell Oxidative Stress Factors

To evaluate the possible effect of biomaterials on the cellular stress, the levels of nitric oxide (NO, Griess Reagent Kit, Thermo Scientific), reactive oxygen species (ROS, CellROX, Thermo Scientific) and superoxide dismutase (SOD, SOD determination kit, Sigma Aldrich) in the conditioned culture media were determined according to the protocols provided by vendors. At least two replicates were performed for each experimental sample.

#### 2.3.14. SEM-EDX Analysis

To evaluate the morphology of the cells cultured on each biomaterial, fixed samples were washed three times in PBS, followed by their dehydration in a graded ethanol series and drying in dessicator. The samples were mounted on microscope stubs, sputtered with gold using a ScanCoat6 coater (Edwards), and observed with a scanning electron microscope (EVO LS15, Zeiss, Oberkochen, Germany) at 10 kV of filament tension. Energy-dispersive X-ray spectrometry (EDX, Quantax Bruker, Billerica, MA, USA) was performed at 20 kV of filament tension.

#### 2.3.15. Quantitative Analysis of Proteins Present in the Conditioned Media

To evaluate the concentration of specific proteins in the collected conditioned media, enzyme-linked immunosorbent assay (ELISA) was used. A complete list of assayed proteins and kits used for the experiment is shown in Table 4. The proteins were detected using monoclonal antibodies conjugated to horseradish peroxidase (HRP). The enzyme substrate consisted of 3,3,5,5-tetramethylbenzidine (TMB). Colorimetric reaction was stopped with 2N sulphuric acid (VI). The absorbance was measured at a single wavelength of 450 nm using a microplate reader.

### 2.4. Statistical Analysis

The data were analysed using one way analysis of variance with Bonferroni post-test. Calculation were made with the Instat 1.14 software (La Jolla, CA, USA).

## 3. Results and Discussion

### 3.1. Microstructure

**Figure 1:**
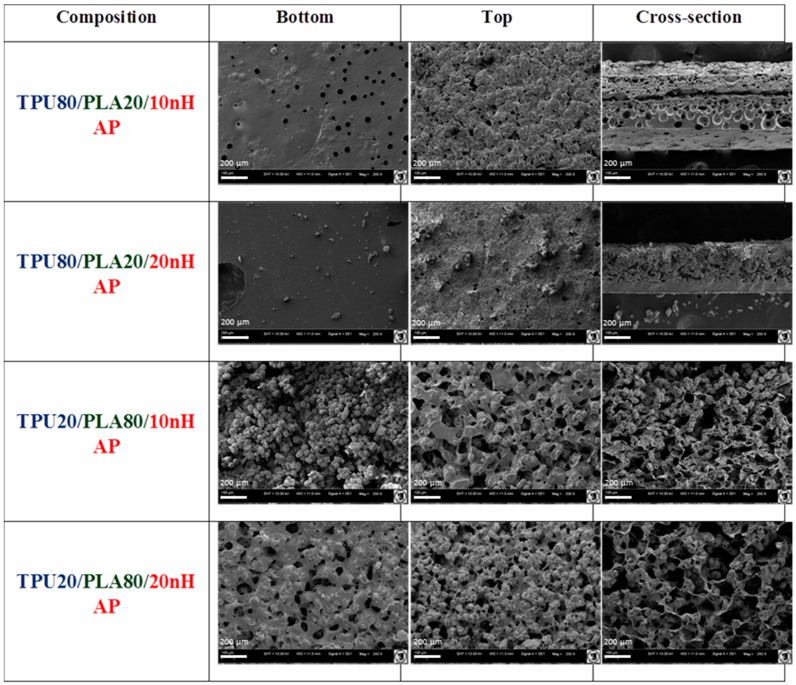
SEM image of the surface and cross-section of TPU/PLA/nHAp films, scale bar = 200 µm.

**Figure 2:**
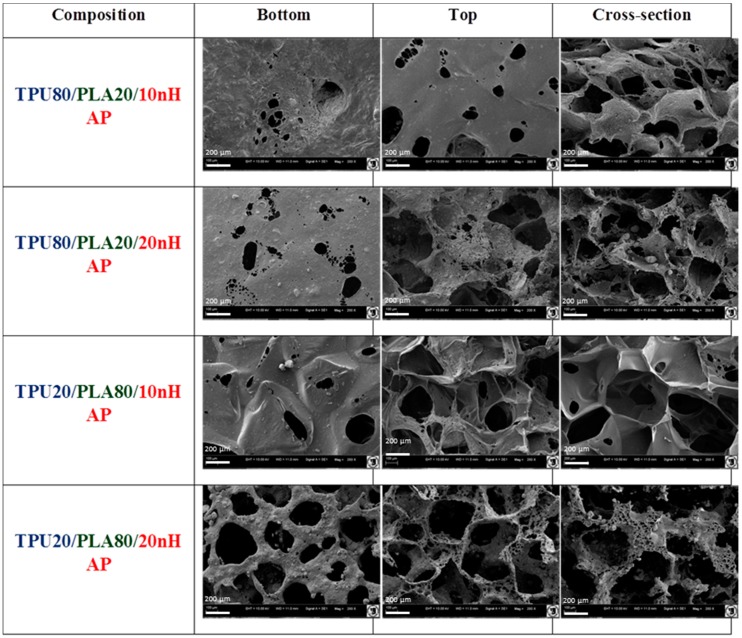
SEM images of the surfaces and cross-sections of TPU/PLA/nHAP sponges, magnification 200×, scale bar = 200 µm.

SEM images of TPU/PLA/HAp composite films and sponges are shown in Figure 1 and Figure 2, respectively. Figure 1 shows that TPU/PLA 8/2 blends with hydroxyapatite nanoparticles exhibited similar surface structure with a low number of visible pores at both 10% and 20%. The higher concentration of nanoHAp resulted in the finest grains visible on the top surface. Bottom surfaces of both TPU/PLA 80/20 blends (10% and 20% of nHAp) were similar to each other, with a relatively smooth surface and only single grains visible. Such topography of polyurethane/polylactide blends has been already observed previously [7]. The second sponge composite—the TPU/PLA 20/80 blend with different concentrations of nanoHAp (10% and 20%) exhibited different topography than the TPU/PLA 8/2 blend; both sides were porous and contained prominent grains. However, the addition of 20% nHAp resulted in finer grains. Cross-sections of the analysed films revealed that a small number of large and numerous small pores were present inside. The differences in surface topography between TPU/PLA 80/20 and 20/80 were caused by different quantities of each component, as described earlier [7].

Figure 2 shows three representative areas on/in the prepared nanocomposite sponge. SEM observation demonstrated microporous structure, which was present in all TPU/PLA-based nanocomposites. It was found that the pores in the composite sponge were rather regularly distributed and rarely interconnected. Interconnectivity was present, but still most of the pores were poorly connected with the adjacent ones. There were no significant differences between the TPU20/PLA 80-based sponge and the TPU80/PLA20-based composite sponge, however, sponges containing more polylactide had the most regular porous structure.

### 3.2. SEM-EDX Analysis

**Figure 3:**
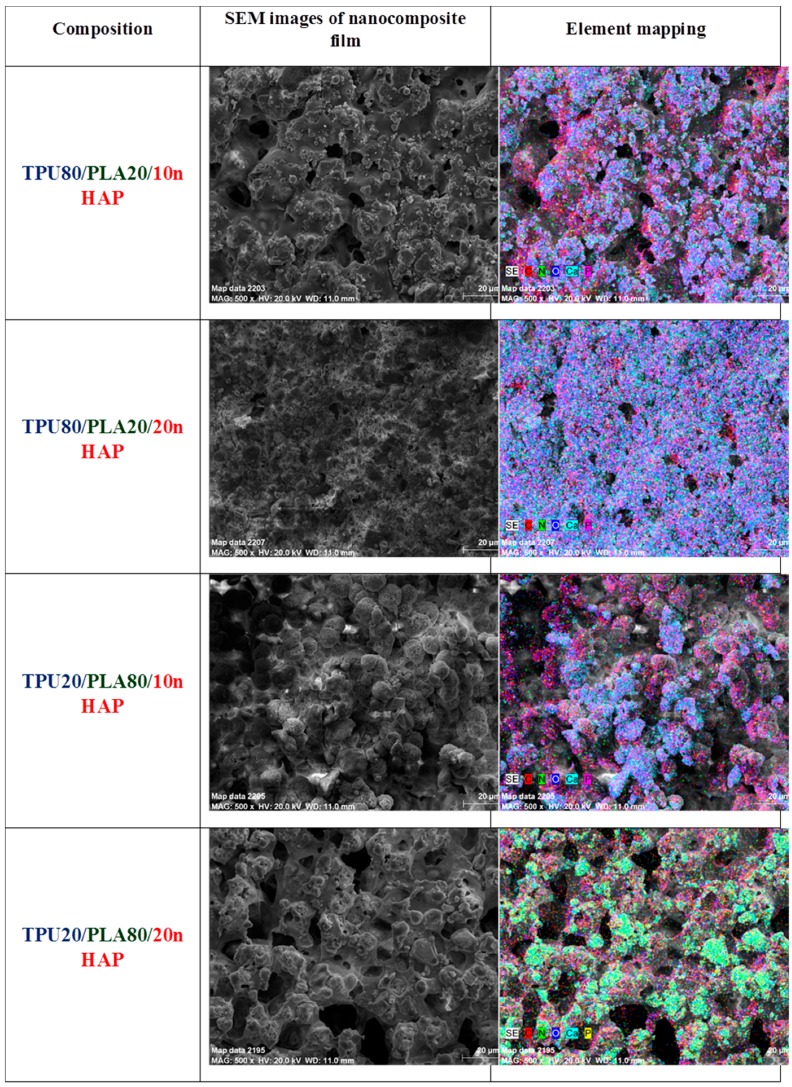
Elemental mapping on the TPU/PLA/nHAP film surface: C (red), N (green), O (blue), Ca (cyan), and P (magenta), magnification 500×.

**Figure 4:**
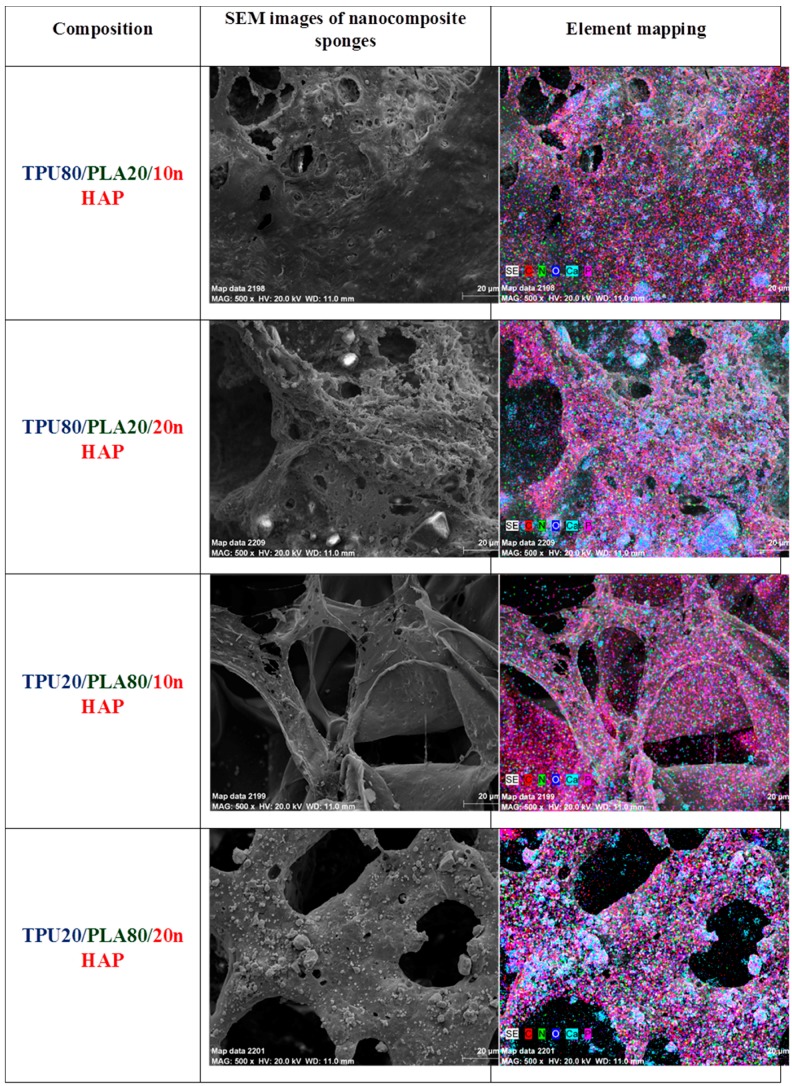
Elemental mapping on the TPU/PLA/nHAP sponge surface: C (red), N (green), O (blue), Ca (cyan), and P (magenta), magnification 500×.

EDX mapping images (SEM images and the corresponding EDX mapping image of the elements: C, O, N, P and Ca) on the surface of TPU/PLA/nHAP films and sponges are presented in Figure 3 and Figure 4, respectively. It can be clearly seen that P and Ca areas overlap, indicating the presence of hydroxyapatite nanoparticles incorporated in the TPU/PLA nonporous and porous blends. Importantly, elemental maps demonstrate that the nHAP particles are regularly dispersed across the polymer nanocomposite surface. As described before, in order to obtain homogenous physical properties of an implant, the distribution of Hap should also be homogenous [20]. While in TPU80/PLA20 its distribution is relatively homogenous, HAp in TPU20/PLA80 was mostly concentrated in polymer grains, especially in the TPU20/PLA80 sample doped with 20% HAp.

### 3.3. Profilometry

In our study, we found that the roughness of the investigated composite films might be increased through the addition of rigid polylactide to elastic polyurethane. Moreover, incorporation of HAp nanoparticles to the TPU/PLA blend increased composite surface roughness (Table 5).

### 3.4. Water Absorption Capacity, Wettability

A series of porous nanocomposite samples of different wettability were tested for water absorption. The fabricated TPU80/PLA20/nHAp nanocomposite sponge exhibited a moderate rate of water adsorption. The highest water absorption capacity was recorded in the pure PLA sample, whereas the lowest value was observed in pure TPU (Table 6). The hydroxyapatite-incorporated TPU20/PLA80 porous blend showed a high absorption capacity (10 times its own weight). This value is considered as a high capacity, while the other types of polymer-based scaffolds did not exhibit such a high water absorbability [21,22]. All the porous matrices demonstrated a good hydrophilicity. The lowest values were recorded for the TPU20/PLA80-based materials. These experimental data correlated very well with our studies regarding the wettability of the TPU/PLA/nHAp film.

The surface wettability of nanocomposite films was analysed in connection with their composition. Table 7 shows the wettability of TPU/PLA/nHAp to distilled water on air. As summarized in Table 7, the contact angle values of TPU20/PLA80-based samples were less than 90° (approx. 65°), indicating good hydrophilicity and wettability of the analysed material. The static water contact angle for clean and pure TPU80/PLA20 blend substrate was 121.7° ± 1.8° (hydrophobic). However, the intercalation of nHAp nanoparticles resulted in a more hydrophilic surface, with a water contact angle lower than 110°. Table 5 demonstrates that the nHAp particles alone could significantly increase the hydrophilicity of the surface. This observation was consistent with the results obtained by Jiang et al. [23], where the addition of nHAp to polycaprolactone-based scaffolds increased their wettability. It is noteworthy that the degree of hydrophobicity also depended on the concentration of polyurethane added to the polymer blend, with a decrease in wettability with an increase in the TPU content. Similar results were obtained previously during the evaluation of polyurethane/polylactide blends without nHAp [7]. Moreover, Kolanthai et al. reported that the wettability could also be modified by the modification of the solvent used for the sol-gel preparation technique [24]. The water contact angles measured on each surface are presented in Table 7.

It could be observed that the value of water contact angle is correlated with the concentration of polylactide and hydroxyapatite nanoparticles High standard deviation values observed in TPU/PLA/nHAp films could indicate that the layer of the composite film was not uniform on the substrate. Increased standard deviation could be associated with an increase in nHAp content, which corresponded with a less uniform distribution of nHAp revealed by SEM-EDX.

### 3.5. Mechanical Properties

The TPU/PLA based blend films were tested for tensile strength and the results are presented in Table 1. The mechanical properties of TPU/PLA blends doped with nHAp were lower in comparison with those of rigid polylactide. Tensile strength of pure PLA was 28.3 MPa, whereas that of TPU/PLA blends doped with nHAp was between 5 and 16 MPa. The nHAp content was mainly responsible for the mechanical properties of TPU-based blends (Table 8).

**Figure 5:**
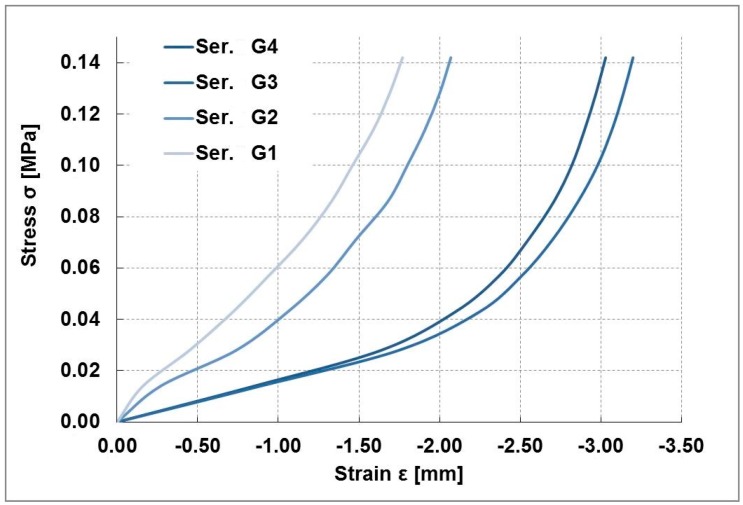
Compressive stress—strain curve for TPU/PLA/nHAp nanocomposite sponges: G1—TPU80/PLA20/nHAp10, G2—TPU/PLA/nHAp20, G3—TPU20/PLA80/nHAp10, and G4—TPU20/PLA80/nHAp20.

The addition of PLA with TPU was chosen to improve the mechanical properties of elastic polyurethane in respect to the Young’s modulus and compressive strength. TPU20/PLA80/nHAp nanocomposites were more rigid than TPU80/PLA20/nHAp. Polyurethane-based blend exhibited a significant increase in the Young’s modulus value with increasing amounts of polylactide and nHAp in the TPU/PLA/nHAp composition (Figure 5). These observations were consistent with previously published results, where the increase in PLA content increased the stiffness of the biomaterial [7]. Moreover, higher nHAp content correlated with a higher Young’s modulus, which is consistent with previous report [21].

Incorporation of HAp nanoparticles significantly increased the compression modulus of the TPU/PLA blend scaffold compared to the sponge without nHAp particles. The compression modulus of the scaffolds increased from 0.9 MPa for pure TPU sponge to 1.3, 1.4, 5.3 and 6.2 MPa for composite scaffolds (TPU/PLA 80/20 + 10 nHAp, TPU/PLA 80/20 + 20 nHAp, TPU/PLA 20/80 + 10 nHAp and TPU/PLA 20/80 + 20 nHAP, respectively). The compression modulus of pure PLA sponge was 7.8 MPa (Table 9).

### 3.6. Thermogravimetric Analysis (TGA) and Differential Scanning Calorimetry (DSC)

**Figure 6:**
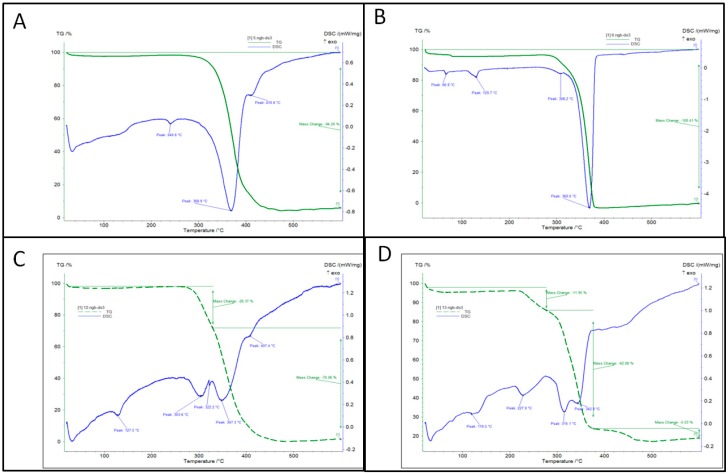
Representative TGA and DSC curves of TPU (**A**); PLA (**B**) and TPU/PLA 80/20 (**C**); TPU/PLA 20/80 (**D**) blends.

Examples of TGA/DSC curves for various formulations of polyurethane-based materials are shown in Figure 6. It is evident that the samples exhibited different thermal degradation behaviour. The temperature range used for the analysis was 20–600 °C. For pure nHAp, dehydration occurred in the whole temperature range of 30–750 °C. Thermoplastic polyurethane started to decompose at a temperature of 300 °C. Figure 6 demonstrates that TPU/PLA blends degraded at a lower temperature than polyurethane or polylactide. It is interesting to note that the degradation temperature of the composite sample was higher than that of pure polymers and their blends. All composite samples containing nHAp showed similar thermal behaviour. However, the sample with an nHAp ratio of 20 wt % had a higher degradation temperature than those containing 10 wt % nHAp.

### 3.7. Focused Ion Beam—Scanning Electron Microscopy

**Figure 7:**
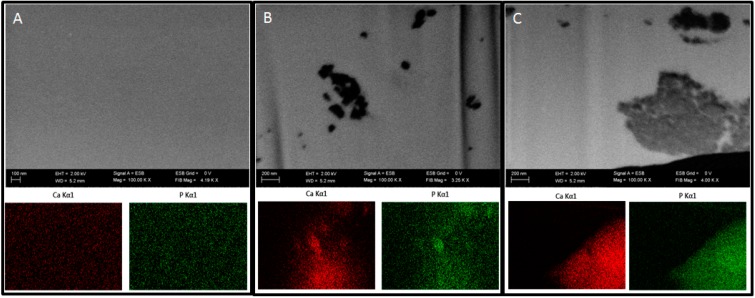

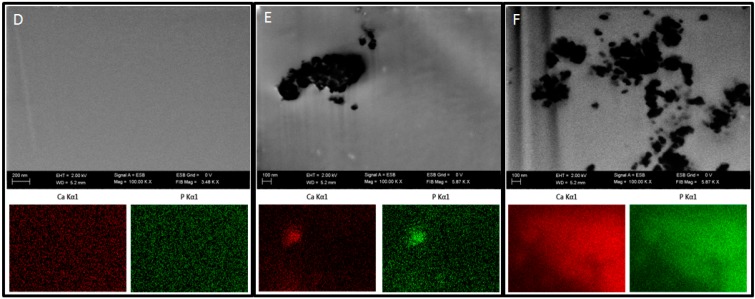
The presence of nHAp particles in the 80/20 ((**A**) no nHAp added; (**B**) 10 wt % of nHAp added; (**C**) 20 wt % of nHAp added) and 20/80 ((**D**) no nHAp added; (**E**) 10 wt % of nHAp added; (**F**) 20 wt % of nHAp added) blends, as revealed by FIB milling and imaging at 2 kV using EsB^®^ detector; colour images demonstrate the distribution of calcium (red) and phosphorus (green) in regions corresponding to SEM images; scale bars on each micrograph indicate 100,000× magnification.

Microscopic analysis revealed uniform distribution of PLA and TPU components within blends. The samples doped with nHAp contained prominent nanoparticles, detected using high magnification. In the samples doped with 10% of nHAp, the particles were rather dispersed, while in the samples doped with 20% of nHAp, a higher level of particle aggregation was observed. SEM-EDX mapping confirmed the presence of calcium and phosphorus in the particles (Figure 7). The use of FIB milling and low voltage polished surface analysis was dictated by the fragility of a diamond knife in ultramicrotome and low chances to cut ultrathin sections with nanoparticles present within. Moreover, ultrathin sections of these polymers are easily damaged at high voltages used in standard TEMs. FIB allowed to obtain chemically unchanged, perfectly polished surfaces for subsequent analysis. The application of EsB detector at low acceleration voltage clearly showed even the slightest differences in the composition [25].

### 3.8. In Vitro Evaluation of the Biomaterial

#### 3.8.1. Flow Cytometry

**Figure 8:**
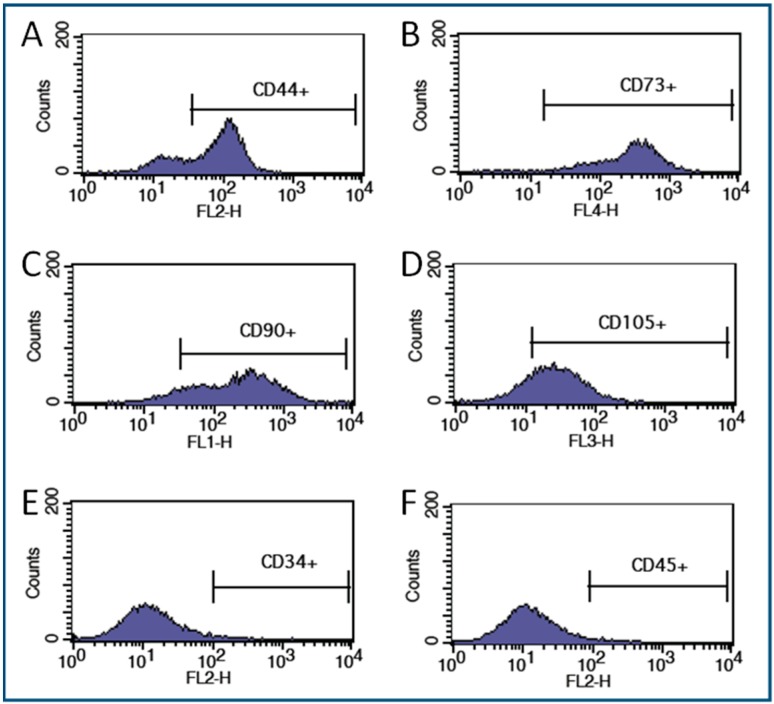
The histograms from flow cytometric analysis showing the presence of CD44 (**A**); CD73 (**B**); CD90 (**C**); CD105 (**D**); and the absence of CD34 (**E**); and CD45 (**F**).

Cells used in the experiment were positive for all mesenchymal-specific surface molecules, CD44, CD73, CD90 and CD105, and negative for hematopoietic lineage-specific proteins, CD34 and CD45 (Figure 8). Therefore, the cells used in the experiment met the minimum criteria defining multipotent stromal stem cells, according to the International Society for Cellular Therapy [26].

#### 3.8.2. Cell Viability and Proliferative Activity

**Figure 9:**
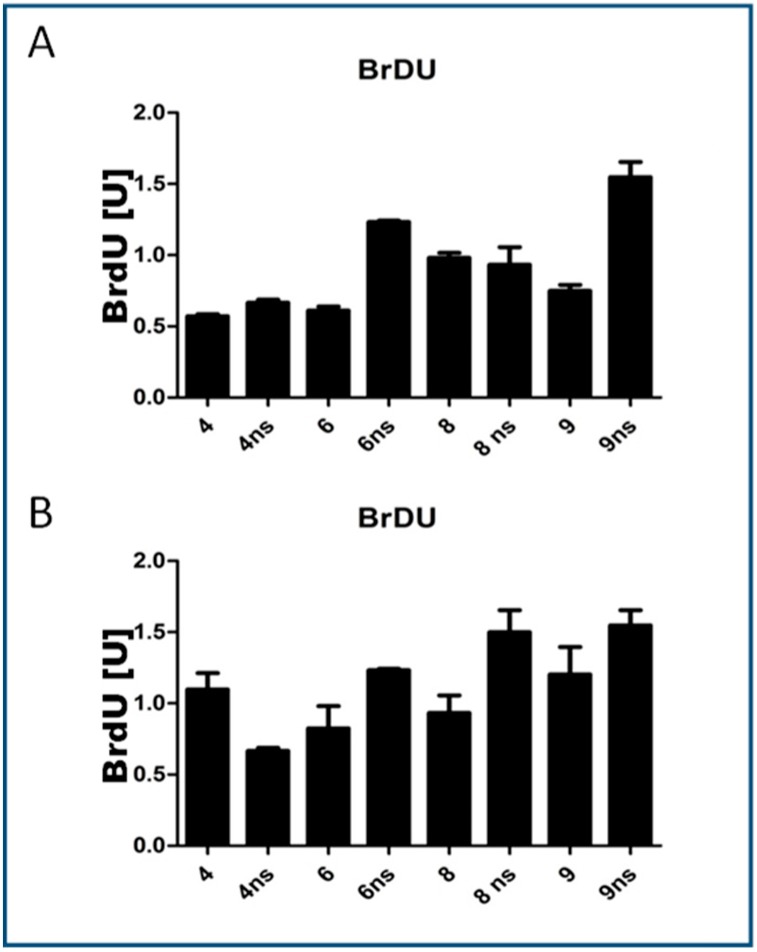
The results of the BrdU assay showing the differences in cell proliferation between experimental groups during the whole experiment ((**A**) chondrogenic culture; (**B**) osteogenic culture, ns—non-stimulated control, materials described in Table 3).

The proliferative activity of the cells cultured on each tested biomaterial in chondrogenic, osteogenic or standard culture media was determined by BrdU incorporation analysis. The analysis showed that the highest proliferative activity was achieved in sample 9, in standard culture medium. Chondrogenic and osteogenic differentiation of the cells caused inhibition of proliferation, with the exception of osteogenic cell culture on biomaterial 4, where the cells showed higher level of proliferation than the non-osteogenic control on the same biomaterial (Figure 9). However, the differences between all the groups were statistically insignificant. Nevertheless, the results clearly indicated that polymer blends used in the experiment did not negatively affect the viability and proliferative activity of cells, as expected based on the previously published results [6,7,27].

#### 3.8.3. Quantitative Analysis of Cellular Oxidative Stress Factors

**Figure 10:**
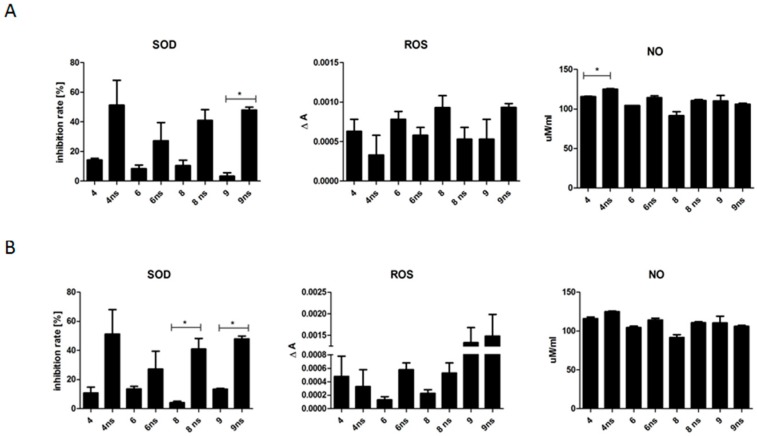
Levels of superoxide dismutase (SOD), reactive oxygen species (ROS) and nitric oxide (NO) in cells cultured in chondrogenic (**A**) and osteogenic (**B**) conditions and in non-stimulated control conditions (ns) in all tested biomaterials (4, 6, 8, 9, described in Table 3); * *p* < 0.05.

The quantitative analysis of cellular stress factors revealed that chondrocyte and osteoblast precursors cultured on tested biomaterials released significantly less superoxide dismutase than the controls non-stimulated towards differentiation. This dependence was visible in all biomaterial samples. In biomaterials 8 and 9, the differences were statistically significant, which suggested the influence of nHAp content, but these samples had different polymer ratios in the blend. The analysis of reactive oxide species in the conditioned media revealed no statistically significant differences between the analysed groups. It is known that hydroxyapatite may induce the formation of reactive oxygen species, however, its cytotoxicity may differ between different cell types, as shown previously [28]. The supernatants of cells cultured on biomaterial 4, 6 and 8 under chondrogenic and osteogenic conditions had lower concentrations of nitric oxide than the respective non-stimulated control groups. However, this difference was statistically significant only in sample 4 in chondrogenic precursor cells (Figure 10). It was reported previously that hydroxyapatite might induce an elevation of nitric oxide in cells; however, this effect was observed only in endothelial cells [29].

#### 3.8.4. SEM-EDX Analysis

**Figure 11:**
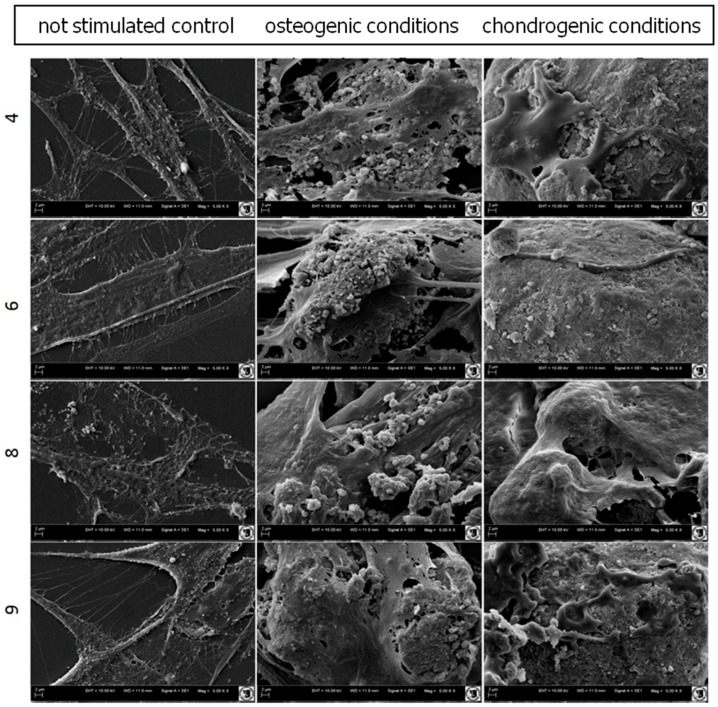
Scanning electron micrographs presenting cells cultured on each biomaterial (4, 6, 8, 9—rows, described in Table 3) at different culture conditions (columns); mag. 5000×, scale bars indicated on micrographs.

**Figure 12:**
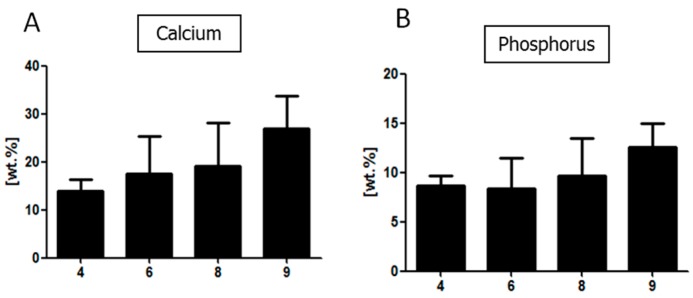
The results from SEM-EDX measurements of calcium and phosphorus concentrations deposited on each biomaterial during osteogenic differentiation (materials described in Table 3).

Microscopic observations showed a differential effect of each biomaterial on the character of ECM deposition by the cells and their morphology. Observations of cells cultured on biomaterials in non-stimulating conditions (control) demonstrated that the cells had a proper fibroblast-like shape with numerous spindle-shaped, bipolar cells observed on biomaterials 4, 6 and 9. Bipolar morphology can be associated with more myogenic phenotype of stromal cells, which could be induced by a higher softness of biomaterials 4 and 9. As already shown, the physical characteristics of the substrate used for cell adhesion strongly influenced the differentiation of stem cells towards one of possible lineages—materials of a moderate stiffness may induce stromal cell differentiation towards myocytes [30]. Moreover, cells developed numerous cytoplasmic processes in these biomaterials, which indicated a high intercellular communication activity. This feature is currently considered as one of the most possible mechanisms of mesenchymal stromal stem cell-based regeneration [31]. Cells cultured in non-stimulating control conditions in biomaterial 8 exhibited degenerative changes, however, the number and size of chondrogenic and osteogenic nodules was the highest of all groups. Since the chondrogenic and osteogenic differentiation involves autophagosomy, the presence of degenerative changes should not be considered as a negative phenomenon [32]. Microscopic observations revealed the presence of round, chondrogenic nodules consisting of cells and ECM. In case of osteogenic differentiation, the nodules that formed in biomaterial 8 were the smallest, but numerous calcium phosphate crystals were observed. On biomaterial 9, the cells developed large osteogenic nodules surrounded by bone-like ECM, with visible processes characteristic of osteoblasts. Despite the proper morphology of the cells grown on biomaterial 4 under non-stimulating control conditions, this biomaterial did not positively influence the process of chondrogenic and osteogenic differentiation. Biomaterial 6 did not stimulate the process of chondrogenic differentiation, however, many cell clusters and calcium phosphate crystals were observed in this group (Figure 11). The measurements of calcium and phosphorus in osteogenic nodules performed with SEM/EDX identified these elements in all treatment groups. However, the highest concentrations of these substances were detected in biomaterials 8 and 9 (Figure 12). This suggested that the presence of nHAp in these samples additionally improved the process of ECM synthesis and deposition, but the mechanism of this action was not clear. Probably nHAp present in the materials served as the core of crystallisation facilitating the deposition of subsequent hydroxyapatite layer, however, it was not verified in this study.

#### 3.8.5. Quantitative Measurements of Proteins from Chondrogenic Cell Media

**Figure 13:**
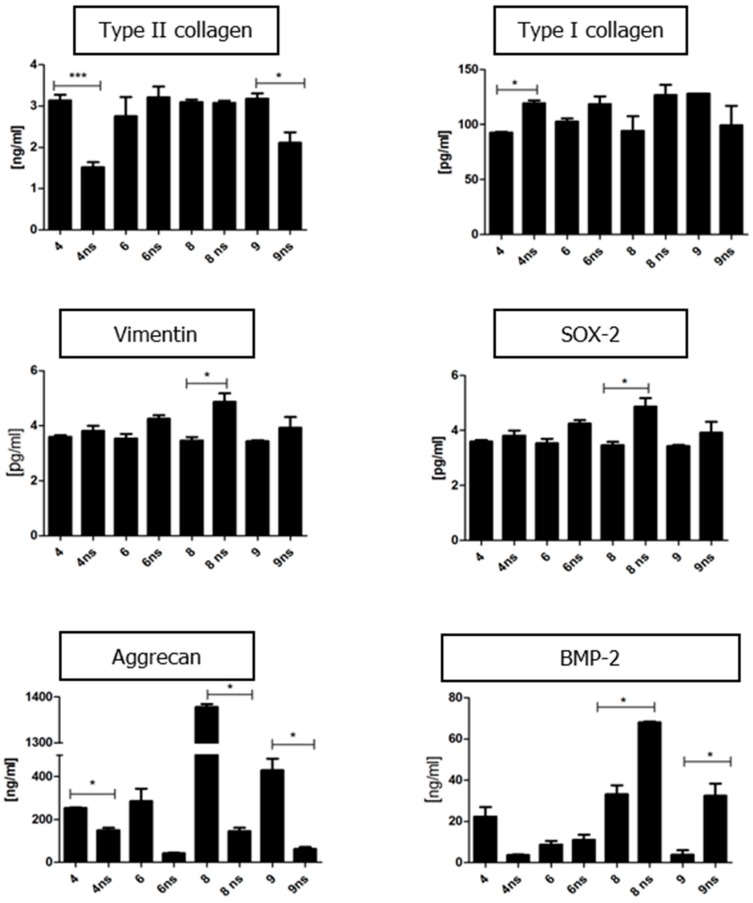
The levels of analysed proteins in chondrogenic and standard (ns) conditions in each tested biomaterial; * *p* < 0.05 (materials described in Table 3).

The conditioned media collected from cell cultures maintained under chondrogenic and standard conditions were analysed for the presence and quantity of type I and II collagens, vimentin, sox-2, aggrecan and bone morphogenetic protein-2 (BMP-2). In biomaterials 4 and 9, chondrogenic differentiation resulted in a statistically significant increase of collagen II production in comparison to the non-stimulated control, which confirmed that they did differentiate towards cartilage tissue cells. However, only in biomaterial 4, the level of collagen I was significantly higher in non-stimulated control than in the chondrogenic group. This corresponded to microscopic observations, where the number of chondrogenic and osteogenic nodules formed on the biomaterial was very low. The level of collagen in the remaining groups was not significantly different. The level of vimentin was significantly elevated only in biomaterial 8 in non-stimulated control in comparison to chondrogenic conditions, and the same situation was recorded for the sox-2 protein. The level of aggrecan was significantly increased in groups under chondrogenic conditions for all biomaterials, except biomaterial 6, where the increase was not statistically significant. The level of BMP-2 was significantly higher in non-stimulated control groups in biomaterials 8 and 9, while in the remaining two biomaterials, the differences were statistically insignificant (Figure 13). Therefore, the level of chondrogenic differentiation was considered as the highest in biomaterials 4 and 9, while the detected levels of proteins most specific for chondrogenesis (collagen II, aggregan) [33] were significantly higher in these two groups in comparison to non-stimulated control. Therefore, we postulate that the most optimal composition for the “cartilage side” of future implant should be produced from TPU80/PLA20, regardless of the nHAp content.

#### 3.8.6. Quantitative Measurements of Proteins from Osteogenic Cell Media

**Figure 14:**
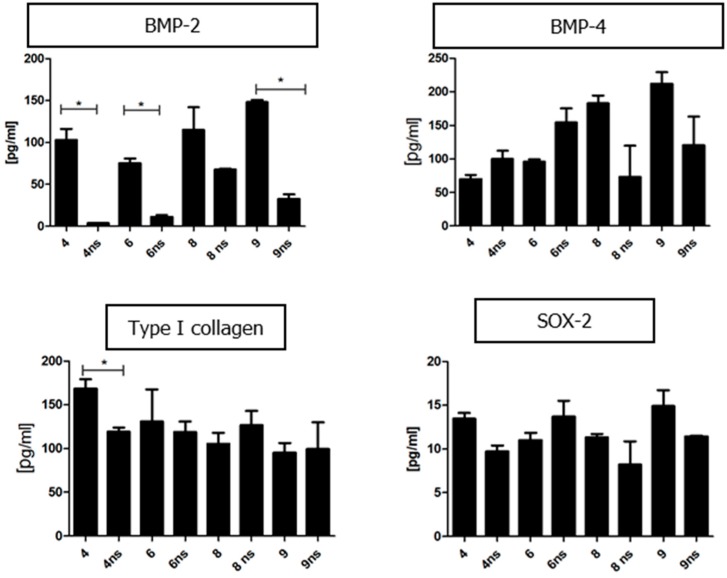
The levels of analysed proteins in the conditioned culture media under osteogenic conditions and standard conditions (non-stimulated control, ns); * *p* < 0.05, *** *p* < 0.001 (materials described in Table 3).

Quantitative measurements of selected proteins revealed that osteogenic culture conditions significantly increased the level of BMP-2 in the conditioned media in all biomaterials, only in biomaterial 8 this increase was statistically insignificant. In addition, in biomaterials 8 and 9, there was an increase in BMP-4 production in osteogenic condition groups. Osteogenic conditions promoted secretion of type I collagen and sox-2 protein by cells cultured on biomaterial 4 in comparison to non-stimulated control. Increased secretion of sox-2 could be also observed in samples 8 and 9 (Figure 14). Similarly to chondrogenic differentiation, the most effective types of surfaces for osteogenic differentiation were samples 4 and 9, based on the levels of collagen I and BMP-2, as the most specific proteins for osteogenesis [34].

## 4. Conclusions

We showed the possibility of synthesizing bi-phasic, polymer-based, nanohydroxyapatite-doped scaffolds for mesenchymal stromal cell colonization and differentiation towards osteoblasts and chondroblasts. These bio-polymer constructs could be considered as a promising tool for bone and cartilage tissue engineering. Of the different types of surfaces, the highest rate of chondrogenic differentiation, was recorded in the samples composed of TPU80/PLA20 with 10% of nHAp, whereas the highest ratio of osteogenic differentiation was noticed in sample TPU20/PLA80 with 20% of nHAp. Therefore, the optimal construction of the scaffold designed for bone and cartilage regeneration is TPU80/PLA20/10nHAp for cartilage side, and TPU20/PLA80/20nHAp for bone side.

## Figures and Tables

**Table 1:** Composition of the polymer nanocomposite films.

Sample name and composition	Mass of PLA (g)	Mass of TPU (g)	DMF volume (mL)	Mass of nHAp (g)
4(TPU80PLA20nHAp10)	1.12	4.48	100	0.175
6(TPU80PLA20nHAp20)	1.12	4.48	100	0.350
8(TPU20PLA80nHAp10)	4.48	1.12	100	0.175
9(TPU20PLA80nHAp20)	4.48	1.12	100	0.350

**Table 2:** Compositions of the porous polymer nanocomposite matrices.

Sample name and composition	Mass of PLA (g)	Mass of TPU (g)	DMF volume (mL)	Mass of nHAP (g)
TPU80PLA20nHAp10	2.24	8.96	100	0.35
TPU80PLA20nHAp20	2.24	8.96	100	0.70
TPU20PLA80nHAp10	8.96	2.24	100	0.35
TPU20PLA80nHAp20	8.96	2.24	100	0.70

**Table 3:** The composition and numbers of biomaterial samples used in the in vitro experiment.

Sample number	Sample composition
4	TPU80/PLA20 + 10 nHAp
6	TPU20/PLA80 + 10 nHAp
8	TPU20/PLA80 + 20 nHAp
9	TPU80/PLA20 + 20 nHAp

**Table 4:** The list of proteins measured in the conditioned media after the last day of the in vitro experiment, and commercial names and producers of assay kits.

Protein	Assay Kit	Producer
Aggrecan	EIAab ELISA Kit	EIAab Science Co. (Wuhan, China)
Type I collagen	EIAab ELISA Kit	EIAab Science Co. (Wuhan, China)
Type II collagen	EIAab ELISA Kit	EIAab Science Co. (Wuhan, China)
Vimentin	EIAab ELISA Kit	EIAab Science Co. (Wuhan, China)
Bone morphogenetic protein-2 (BMP-2)	Quantikine^TM^ ELISA	R&D Systems (Minneapolis, MN, USA)
Bone morphogenetic protein-4 (BMP-4)	Quantikine^TM^ ELISA	R&D Systems (Minneapolis, MN, USA)
SOX-2	ELISA Kit	MyBioSource (San Diego, CA, USA)

**Table 5:** The analysis of the experimental surface roughness determined in all composite materials and pure TPU and PLA films. *p*-values shows the statistical significance between the series (*p* < 0.05).

Sample code	Content of nHAp (wt %)	Roughness (*R*_a_) (μm)	*p*-Value
**TPU (Elastic)**	-	0.3 ± 1.9	*p* < 0.05
**TPU/PLA 80/20**	-	5.4 ± 1.2	*p* < 0.05
**TPU/PLA 80/20**	10	6.3 ± 2.1	*p* < 0.05
**TPU/PLA 80/20**	20	7.4 ± 2.6	*p* < 0.05
**TPU/PLA 20/80**	10	7.2 ± 1.8	*p* < 0.05
**TPU/PLA 20/80**	20	8.1 ± 1.6	*p* < 0.05
**TPU/PLA 80/20**	-	5.9 ± 1.5	*p* < 0.05
**PLA (Rigid) [7]**	-	1.26 ± 0.93	*p* < 0.05

**Table 6:** The analysis of water adsorption of the prepared composite materials and pure TPU and PLA films.

Sample code	Content of nHAp (wt %)	Water adsorption (%)
**TPU (Elastic)**	-	325 ± 8
**TPU/PLA 80/20**	-	492 ± 13
**TPU/PLA 80/20**	10	524 ± 12
**TPU/PLA 80/20**	20	568 ± 9
**TPU/PLA 20/80**	10	781 ± 15
**TPU/PLA 20/80**	20	825 ± 22
**TPU/PLA 80/20**	-	756 ± 27
**PLA (Rigid)**	-	941 ± 10

**Table 7:** Static water contact angle measurements for different TPU/PLA/nHAP nanocomposite and TPU/PLA blend surfaces.

Sample code	Static contact angle (°)
**Pure TPU (control)**	123.2 ± 1.8
**Pure PLA (control)**	79.9 ± 1.1
**5STPU80/PLA20 (control)**	121.7 ± 1.8
**5STPU20/PLA80 (control)**	63.4 ± 1.5
**5STPU80PLA20nHAp10**	105.1 ± 1.2
**5STPU80PLA20nHAp20**	100.2 ± 0.9
**5STPU20PLA80nHAp10**	62.25 ± 1.4
**5STPU20PLA80nHAp20**	69.1 ± 1.7

**Table 8:** Mechanical properties of the blend of TPU and PLA doped with nano-hydroxyapatite in comparison with pure polyurethane and polylactide—tensile test.

Sample code	Content of nHAp (wt %)	Tensile strength σ (MPa)	Elongation break (%)
**TPU (Elastic)**	-	3.9 ± 0.6	36.3 ± 2.4
**TPU/PLA 80/20**	-	5.1 ± 0.5	28.7 ± 3.1
**TPU/PLA 80/20**	10	5.3 ± 1.4	9.7 ± 1.8
**TPU/PLA 80/20**	20	5.7 ± 1.9	9.9 ± 2.1
**TPU/PLA 20/80**	10	14.7 ± 1.3	7.5 ± 1.2
**TPU/PLA 20/80**	20	15.5 ± 2.2	7.3 ± 1.5
**TPU/PLA 20/80**	-	24.0 ± 1.8	8.1 ± 1.4
**PLA (Rigid)**	-	28.3 ± 1.3	6.4 ± 1.7

**Table 9:** Mechanical properties of the blend of TPU and PLA doped with nano-hydroxyapatite in comparison with pure polyurethane and polylactide—compressive test.

Sample code	Content of nHAp (wt %)	σ (MPa)
**TPU (Elastic)**	-	0.9 ± 0.1
**TPU/PLA 80/20**	-	1.0 ± 0.2
**TPU/PLA 80/20**	10	1.3 ± 0.2
**TPU/PLA 80/20**	20	1.4 ± 0.2
**TPU/PLA 20/80**	10	5.3 ± 0.1
**TPU/PLA 20/80**	20	6.2 ± 0.3
**TPU/PLA 20/80**	-	5.8 ± 0.4
**PLA (Rigid)**	-	7.8 ± 0.8

## Abbreviations

The following abbreviations were used in this manuscript:

nHAp	nano-hydroxyapatite
OCD	osteochondral defects
PU	polyurethane
PLDL	polylactide
OECs	olfactory ensheathing cells
ASCs	adipose stem cells
MSCs	mesenchymal stem cells
MVs	microvesicles
TGF	transforming growth factor
BMP-2	bone morphogenetic protein-2
TPU	thermoplastic polyurethane
PLA	inelastic polylactide
DMF	dimethylformamide
SCSL	solvent casting/salt leaching
HBSS	Hank’s balanced salt solution
FBS	fetal bovine serum
PSA	penicillin/streptomycin/amphotericin b
FACS	fluorescence-activated cell sorter
PBS	phosphate buffered saline
BrdU	bromodeoxyuridine
NO	nitric oxide
ROS	reactive oxygen species
SOD	superoxide dismutase
HRP	horseradish peroxidase
TMB	3,3,5,5-tetramethylbenzidine (TMB)
ECM	extracellular matrix
